# Adjunctive electroacupuncture to facilitate discontinuation of non-benzodiazepine hypnotics in chronic insomnia: a randomized controlled trial protocol

**DOI:** 10.3389/fneur.2026.1835295

**Published:** 2026-05-06

**Authors:** Yaoxin Chen, Pei Shen, Gongyue Zhou, Wenlin Xu, Yijia Wan, Cong Wang

**Affiliations:** 1 Department of Acupuncture and Moxibustion, The First Affiliated Hospital of Zhejiang Chinese Medical University (Zhejiang Provincial Hospital of Chinese Medicine), Hangzhou, China; 2 The First School of Clinical Medicine, Zhejiang Chinese Medical University, Hangzhou, China; 3 Department of Massage, The First Affiliated Hospital of Zhejiang Chinese Medical University (Zhejiang Provincial Hospital of Chinese Medicine), Hangzhou, China

**Keywords:** adjunctive, chronic insomnia disorder, electroacupuncture, non-benzodiazepines, randomized controlled trial

## Abstract

**Background:**

Acupuncture has demonstrated beneficial effects on sleep outcomes in patients with chronic insomnia disorder (CID); however, robust evidence from randomized controlled trials evaluating its efficacy as an adjunctive intervention to facilitate discontinuation of non-benzodiazepines (NBZDs) remains limited.

**Objective:**

To evaluate the clinical efficacy and safety of electroacupuncture as an adjunctive therapy for reducing NBZDs use in patients with chronic insomnia disorder.

**Methods:**

This single-blind, randomized, controlled trial will enroll 78 adults with chronic insomnia disorder (ICSD-3TR) who have used NBZDs for >6 months. Participants will be assigned 1:1 to electroacupuncture or sham electroacupuncture. Both groups will undergo a standardized, gradual NBZDs tapering protocol over 6 weeks and receive standardized sleep hygiene education. Participants in the electroacupuncture group will receive electroacupuncture treatment three times per week at predefined acupoints (Baihui, Sishencong, Anmian, Shenting, Yintang, Hegu, Taichong, Neiguan, Shenmen, Sanyinjiao, Shenmai, and Zhaohai), whereas participants in the control group will receive non-penetrative sham electroacupuncture. The primary outcome is the successful discontinuation rate of NBZDs at the end of the 6-week treatment period. Secondary outcome measures included subjective sleep scales, objective sleep parameters (polysomnography and actigraphy), and daytime functioning assessments, among others. Safety will be assessed through systematic monitoring and documentation of adverse events. Statistical analyses will follow the intention-to-treat principle using mixed-effects models.

**Conclusion:**

This trial aims to provide high-quality evidence regarding the efficacy and safety of electroacupuncture as an adjunctive intervention for NBZDs tapering in patients with chronic insomnia. The findings may support the integration of a low-risk, non-pharmacological treatment option into clinical practice and address an important gap in current insomnia management strategies.

**Clinical trial registration:**

https://itmctr.ccebtcm.org.cn/mgt/project/view/1998715224785551360, ITMCTR2025002446.

## Introduction

Chronic insomnia disorder (CID) is characterized by persistent difficulties in initiating or maintaining sleep, occurring at least three times per week for a duration of no less than 3 months, and accompanied by clinically significant daytime impairment (1). With an estimated prevalence of 25.0% among Chinese adults (2), CID represents the most prevalent sleep disorder in China. It is commonly associated with fatigue, cognitive impairment, emotional dysregulation, and depressive symptoms, leading to substantial impairments in quality of life and functional capacity (3). Pharmacological therapy remains a cornerstone of CID management, with approximately one-fifth of patients relying on sedative–hypnotic medications to initiate or maintain sleep (4). Benzodiazepine receptor agonists (BZRAs), including both benzodiazepines (BZDs) and non-benzodiazepines (NBZDs), are the most frequently prescribed agents. These medications exert their hypnotic effects primarily by enhancing gamma-aminobutyric acid (GABA)–mediated inhibitory neurotransmission, resulting in central nervous system suppression and subsequent sedative and anxiolytic effects (5).

However, long-term BZRAs use is associated with tolerance development and a range of adverse outcomes, including daytime somnolence, cognitive impairment (6), increased risk of falls (7), motor vehicle accidents (8), and elevated all-cause mortality (9). In response to growing concerns regarding dependence and misuse, the U.S. Food and Drug Administration mandated updated boxed warnings in 2020 highlighting the risks of physical dependence, withdrawal reactions, abuse, and addiction associated with BZRAs use (10). Despite these warnings, patient awareness of the dependence potential and withdrawal risks of BZRAs remains limited, contributing to a low spontaneous discontinuation rate of approximately 6% (11).

Various strategies have been explored to facilitate safe and effective BZRAs discontinuation. Pharmacological substitution approaches, although commonly employed, may provoke adverse effects such as panic symptoms (12), increase fall risk (13, 14), or anticholinergic side effects including dry mouth and dysgeusia (15). Moreover, individualized and standardized protocols for BZRAs dose conversion and tapering remain inadequately established (16). In the absence of medications specifically approved by the U.S. Food and Drug Administration or the China National Medical Products Administration for BZRAs dependence or withdrawal, non-pharmacological approaches have attracted increasing attention. Acupuncture has been incorporated into the most recent Chinese clinical guidelines for the diagnosis and treatment of insomnia as a recommended therapeutic option (17, 18). As a widely utilized complementary therapy for insomnia, acupuncture has demonstrated efficacy in improving total sleep time, sleep efficiency, and both subjective and objective sleep quality, while also alleviating cognitive impairment and mood disturbances, with a favorable safety profile (19, 20).

More recently, interest has emerged in the potential role of acupuncture as an adjunctive intervention during BZRAs tapering. A randomized controlled trial by Yeung et al. found no significant difference between electroacupuncture and sham acupuncture in discontinuation rates or withdrawal symptom severity during a four-week tapering protocol (21). Subsequent methodological analyses suggested that the inclusion of both BZDs and NBZDs within the same cohort may have confounded the findings, as different BZRAs subtypes are associated with distinct pharmacokinetic profiles and withdrawal trajectories (22).

NBZDs, such as zolpidem, zopiclone, and zaleplon, are currently recommended as first-line pharmacological treatments for insomnia in many clinical guidelines owing to their shorter elimination half-lives, lower residual sedation, and comparatively reduced dependence potential relative to traditional BZDs (23). Accordingly, NBZDs have become the most commonly prescribed hypnotics in China. Notably, a recent clinical trial reported that electroacupuncture significantly alleviated withdrawal symptoms, improved sleep architecture, increased deep sleep duration, and reduced medication dependence in patients with chronic insomnia undergoing zolpidem tapering (24). The divergent findings of existing studies suggest that acupuncture may exert differential effects during NBZDs discontinuation specifically, rather than across all BZRAs subtypes.

Taken together, current evidence regarding acupuncture as an adjunctive therapy for hypnotic tapering remains limited and inconsistent, particularly with respect to NBZDs. Therefore, this single-blind, randomized, sham-controlled trial is designed to evaluate the efficacy and safety of electroacupuncture as an adjunctive intervention for NBZDs tapering in patients with chronic insomnia disorder. By systematically assessing medication discontinuation rates, sleep quality, daytime functioning, and withdrawal-related outcomes, this study aims to provide robust evidence to inform clinical decision-making in the management of long-term hypnotic use.

## Methods

### Study design

This study is a single-center, randomized, controlled, single-blind clinical trial conducted at the Department of Acupuncture and Moxibustion, Zhejiang Provincial Hospital of Traditional Chinese Medicine. The trial period is scheduled from September 2025 to December 2026. Eligible participants will be randomly assigned in a 1:1 ratio to either the electroacupuncture group or the sham electroacupuncture group using a centralized randomization system.

**Figure 1:**
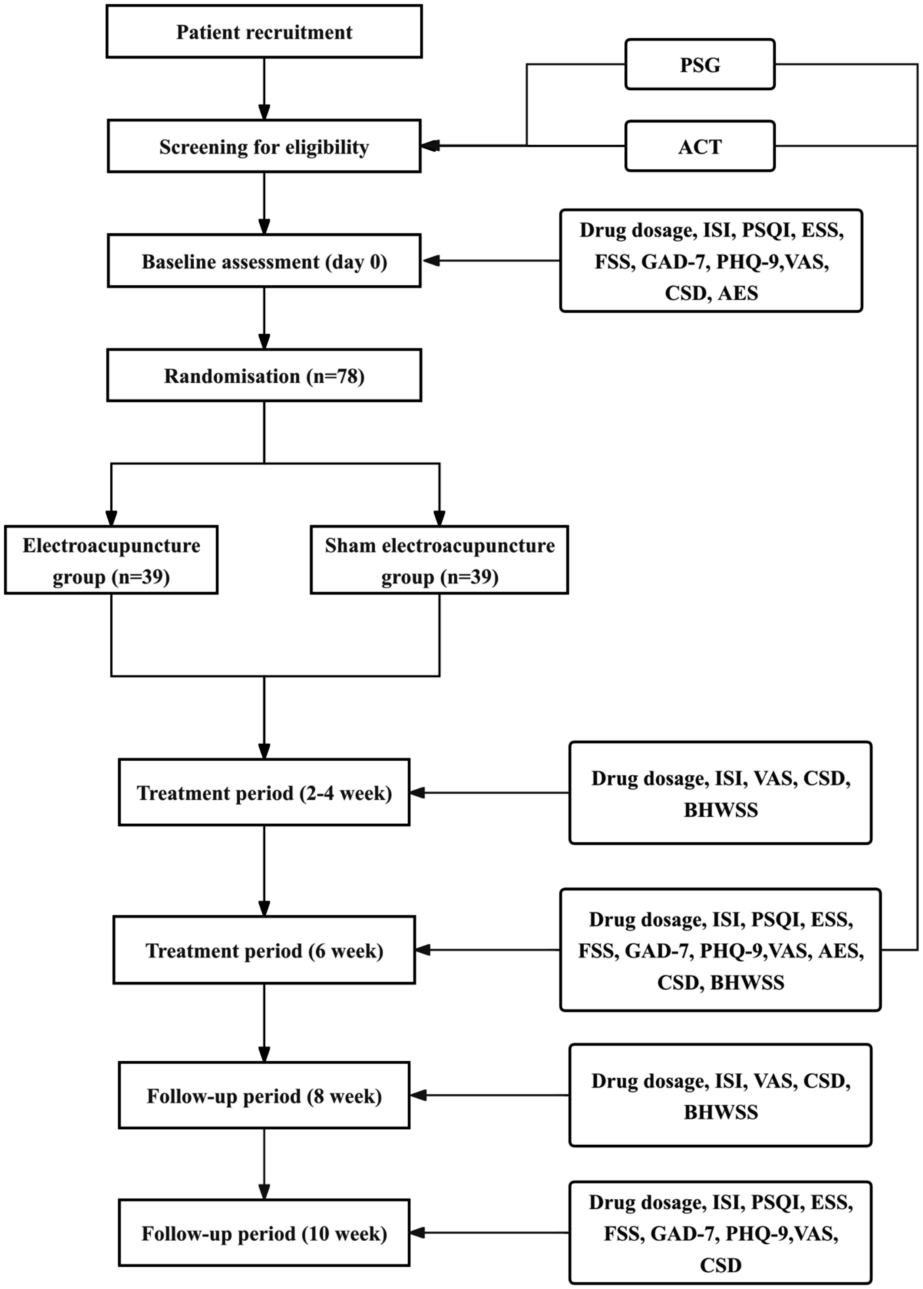
Flow diagram. ACT, actigraphy; AES, Acupuncture Expectancy Scale; BHWSS, Benzodiazepine Hypnotics Withdrawal Symptom Scale; CSD, Consensus Sleep Diary; ESS, Epworth Sleepiness Scale; FSS, Fatigue Severity Scale; GAD-7, Generalized Anxiety Disorder 7-item; ISI, Insomnia Severity Index; NBZDs, Non-Benzodiazepines; PHQ-9, Patient Health Questionaire-9; PSG, polysomnography; PSQI, Pittsburgh Sleep Quality Index; VAS, Visual Analogue Scale; 5-HT: 5-hydroxytryptamine.

A total of 78 participants will be enrolled, with 39 participants allocated to each group. Each participant will undergo three study phases: a screening period, a 6-week treatment period, and a follow-up period, with an overall study duration of approximately 12–13 weeks (Figure 1). The study protocol has been approved by the Ethics Committee of Zhejiang Provincial Hospital of Traditional Chinese Medicine (Approval No.: 2025-KLS-532-01) and will be conducted in accordance with the Consolidated Standards of Reporting Trials (CONSORT) guidelines and the Standards for Reporting Interventions in Clinical Trials of Acupuncture (STRICTA). The study flowchart and procedure are depicted in Figure 1 and Table 1.

**Table 1:** Trial processes chart.

Week	0	1	2	3	4	5	6	7	8	9	10
	Baseline	Treatment phase						Follow-up phase			
Patients											
Enrolment	**×**										
Sign informed consent	**×**										
General information	**×**										
Medication diary	**×**	**×**	**×**	**×**	**×**	**×**	**×**	**×**	**×**	**×**	**×**
PSG	**×**						**×**				
ACT	**×**						**×**				
Groups											
Acupuncture group		18 treatments									
Sham- acupuncture group		18 treatments									
Outcome measurement											
Successful drug discontinuation rate							**×**				
CSD	**×**		**×**		**×**		**×**		**×**		**×**
ISI	**×**		**×**		**×**		**×**		**×**		**×**
PSQI	**×**						**×**				**×**
ESS	**×**						**×**				**×**
FSS	**×**						**×**				**×**
GAD-7	**×**						**×**				**×**
PHQ-9	**×**						**×**				**×**
NBZDs reduction rate	**×**		**×**		**×**		**×**		**×**		**×**
BHWSS			**×**		**×**		**×**		**×**		**×**
VAS	**×**		**×**		**×**		**×**		**×**		**×**
AES	**×**										
Blinding assessment		**×**		**×**			**×**				
Adverse events											
Reasons for dropouts or withdrawals	**×**	**×**	**×**	**×**	**×**	**×**	**×**	**×**	**×**	**×**	**×**

ACT, actigraphy; AES, Acupuncture Expectancy Scale; BHWSS, Benzodiazepine Hypnotics Withdrawal Symptom Scale; CSD, Consensus Sleep Diary; ESS, Epworth Sleepiness Scale; FSS, Fatigue Severity Scale; GAD-7, Generalized Anxiety Disorder 7-item; ISI, Insomnia Severity Index; NBZDs, non-benzodiazepines; PHQ-9, Patient Health Questionaire-9; PSG, polysomnography; PSQI, Pittsburgh Sleep Quality Index; VAS, Visual Analogue Scale; 5-HT, 5-hydroxytryptamine.

### Participants

Participants will be recruited primarily from patients with chronic insomnia disorder who attend the Department of Acupuncture and Moxibustion at Zhejiang Provincial Hospital of Traditional Chinese Medicine and who report long-term use of NBZDs.

The diagnosis of chronic insomnia disorder will be established according to the International Classification of Sleep Disorders, Third Edition, Text Revision (ICSD-3TR), published by the American Academy of Sleep Medicine in 2023 (1).

### Inclusion criteria

Participants must meet all of the following criteria:

(1) Aged 18–65 years, regardless of sex;
(2) Meeting the diagnostic criteria for chronic insomnia according to ICSD-3TR;
(3) Participants were required to have insomnia symptoms of mild to moderate severity (Insomnia Severity Index ≤ 21) while taking NBZDs (25);
(4) Continuous use of NBZDs for chronic insomnia for more than 6 months, with medication adherence exceeding 50% of the treatment period and a dosing frequency of at least three nights per week during the 2 weeks prior to screening;
(5) Use of NBZDs recommended in the Chinese Guideline for the Diagnosis and Treatment of Insomnia in Adults (2023 edition), with each dose not exceeding the guideline-recommended maximum (18);
(6) Self-reported history of at least one unsuccessful attempt to discontinue NBZDs independently;
(7) A clear willingness to reduce NBZDs dosage;
(8) No history of acupuncture or other needle-based therapies within the past 6 months;
(9) Ability and willingness to comply with the study protocol and provision of written informed consent.

### Exclusion criteria

Participants meeting any of the following criteria will be excluded:

(1) Polysomnography (PSG) findings indicating an apnea-hypopnea index ≥15 events/h, a periodic limb movement index with arousal ≥15 events/h, rapid eye movement (REM) sleep without atonia, or evidence of dream enactment behavior (26).
(2) Engagement in shift work within 2 weeks prior to screening or during the study period, or planned travel across more than two time zones.
(3) Use of anxiolytics, antidepressants, antipsychotics, melatonin, or melatonin receptor agonists within 2 weeks prior to screening or during the study period.
(4) History of alcohol or substance use disorder within the past year.
(5) History of severe psychiatric disorders (e.g., bipolar disorder or major depressive disorder).
(6) Severe cardiovascular, hepatic, renal, hematological, or respiratory disease, or uncontrolled infectious disease.
(7) Aversion to acupuncture, needle phobia, or inability to receive acupuncture due to infection, inflammation, scarring, or trauma at or near the selected acupoints.
(8) Pregnancy or breastfeeding.
(9) Participation in another interventional clinical trial within the past 6 months.

### Withdrawal criteria

Participants will be withdrawn from the study if any of the following occur:

(1) Participants who develop intolerable severe withdrawal symptoms during the NBZDs tapering process.
(2) Voluntary withdrawal at the participant’s request.
(3) Withdrawal of informed consent.
(4) Emergence of exclusion criteria during the study period.
(5) Poor treatment compliance, defined as attendance at fewer than 50% of scheduled acupuncture sessions.
(6) Occurrence of adverse events that preclude continued participation or pose a risk to participant safety.

### Interventions

All acupuncture procedures will be performed by a licensed acupuncturist holding a master’s degree in acupuncture and massage from Zhejiang Chinese Medical University, with more than 8 years of formal training and at least 3 years of independent clinical practice. Prior to trial initiation, all study personnel will undergo standardized training to ensure protocol adherence and procedural consistency.

### Electroacupuncture group

Participants in the electroacupuncture group will receive electroacupuncture treatment in addition to a standardized NBZDs tapering protocol.

### NBZDs tapering protocol

Based on the 2025 European clinical practice guideline recommending a gradual dose reduction of 10–25% per week for NBZDs (27), and supported by two clinical studies that successfully employed a weekly 25% reduction strategy (21, 28), NBZDs will be reduced at a rate of 25% of the baseline dose per week. If participants experience intolerable withdrawal symptoms during tapering—such as exacerbation of insomnia, anxiety, or agitation—the dose will be temporarily reverted to the previous level. Tapering will be reattempted during the subsequent evaluation cycle (1 week per cycle).

Throughout the treatment period, participants will attend weekly in-person consultations and receive twice-weekly telephone follow-ups. Dose adjustments will be conducted through a shared decision-making process, enabling participants to actively engage in symptom monitoring and self-management under physician supervision. Final dosage modifications will require investigator approval.

### Acupuncture procedure

Participants will be treated in the supine position, with bilateral acupoint selection at each session. Acupoints will include Baihui (GV20), Sishencong (EX-HN1), Bilateral Anmian (Extra), Shenting (GV24), Yintang (GV24+), Bilateral Hegu (LI4), Bilateral Taichong (LR3), Bilateral Neiguan (PC6), Bilateral Shenmen (HT7), Bilateral Sanyinjiao (SP6), Bilateral Shenmai (BL62), and Bilateral Zhaohai (KI6) (29, 30).

Acupoints will be localized in accordance with the Nomenclature and Location of Acupoints (GB/T 12346–2021), detailed locations are presented in Table 2 (31). After routine skin disinfection with 75% medical alcohol, sterile disposable acupuncture needles (0.25 × 40 mm; Andy Medical Instruments Co., Ltd., Guizhou, China) will be inserted. Manual manipulation will be applied to elicit the deqi sensation, characterized by soreness, numbness, heaviness, or distension. Electrodes will be connected between Shenting–Baihui, bilateral Sishencong, and Shenmai–Zhaohai using a Yindi KWD-808I electroacupuncture device. A continuous wave at 4 Hz will be applied, with intensity adjusted to participant tolerance. Needles will be retained for 30 min.

**Table 2:** Acupoint locations.

Acupoint	Location
Baihui (GV20)	5 cun directly above the midpoint of the anterior hairline on the head
Sishencong (EX-HN1)	At the vertex of the head, 1 cun anterior, posterior, and lateral to Baihui (GV20), four points in total
Anmian (Extra)	Posterior to the ear, in the depression between the sternocleidomastoid and trapezius muscles
Shenting (GV24)	0.5 cun directly above the midpoint of the anterior hairline on the head
Yintang (GV24+)	In the depression at the midpoint between the medial ends of the two eyebrows
Hegu (LI4)	On the dorsum of the hand, between the 1st and 2nd metacarpal bones, at the midpoint of the radial side of the second metacarpal bone
Taichong (LR3)	On the dorsum of the foot, in the depression distal to the junction of the first and second metatarsal bones
Neiguan (PC6)	On the palmar aspect of the forearm, 2 cun proximal to the wrist crease, between the tendons of palmaris longus and flexor carpi radialis
Shenmen (HT7)	At the ulnar end of the transverse crease of the wrist, in the depression on the radial side of the tendon of muscle flexor carpi ulnaris
Sanyinjiao (SP6)	On the tibial aspect of the leg, 3 cun proximal to the tip of the medial malleolus, on the posterior border of the tibia
Shenmai (BL62)	On the lateral side of the foot, in the depression directly below the tip of the lateral malleolus
Zhaohai (KI6)	On the medial side of the foot, in the depression on the inferior border of the medial malleolus, vertically below the apex of the medial malleolus

Treatment will be administered three times per week on alternating days for 6 consecutive weeks. Standardized medication tapering interviews will be conducted prior to weekly acupuncture sessions.

### Sham electroacupuncture group

Participants in the sham electroacupuncture group will receive non-penetrating sham acupuncture in combination with the same standardized NBZDs tapering protocol. Participants will be treated in the supine position using the same acupoint locations as in the electroacupuncture group. After skin disinfection, retractable blunt-tip needles (0.25 × 40 mm; Shanghai Hongman Technology Co., Ltd., China) will be placed within a spacer and tapped to simulate needle insertion without skin penetration. This procedure produces a pricking sensation comparable to real acupuncture while avoiding tissue penetration.

Needles will be connected to the same electroacupuncture device; however, no electrical current will be delivered, despite activation of the device indicator light. Needles will remain in place for 30 min without current adjustment. Treatment frequency and duration will be identical to those of the electroacupuncture group.

### Concomitant medication

Throughout the study period, participants will not be permitted to use any additional pharmacological treatments that may affect sleep, mood, or autonomic function, including antidepressants, anxiolytics, antipsychotics, melatonin, and melatonin receptor agonists.

Medications prescribed for stable chronic medical conditions (e.g., hypertension, diabetes, or dyslipidemia) will be allowed, provided that the dosage has remained unchanged for at least 4 weeks prior to enrollment and throughout the study period.

Any temporary or emergency medication use will be documented in detail, including the drug name, dosage, duration, and indication, and will be considered in subsequent analyses if necessary.

### Outcome measures

#### Primary outcome measures

The successful discontinuation rate at the end of the 6-week treatment was defined as the proportion of patients who had completely ceased NBZDs use (any dose) during the final week of treatment (days 36–42). Medication adherence was verified using patients’ daily diaries and medication records. Patients completed a daily diary card, on which they recorded the date, medication name, dose, time of administration, and any adverse reactions. The diary cards were returned to the physician at each weekly visit, and prescription records were cross-checked by an independent observer every 3 weeks. Any discrepancy between the diary and medication records will be resolved by consensus of two blinded independent investigators based on all available data (daily diaries, medication records). Urine or blood drug concentration testing was not performed to confirm participants’ self-report of hypnotic discontinuation, due to the short half-life of NBZDs (requiring daily sampling, which is logistically infeasible) and the increased participant burden that would likely raise dropout rates. The multi-source adjudication approach described above was considered a reliable alternative.

#### Secondary outcome measures

Secondary outcomes are assessed at baseline, during treatment, and during follow-up. Detailed assessment procedures are provided in the Supplementary Appendix S1; assessment time points are summarized in Table 1.

(1) *Subjective Sleep Scales*

① Consensus Sleep Diary (CSD): Records total sleep time (TST), sleep onset latency (SOL), wake after sleep onset (WASO), sleep efficiency (SE), subjective sleep quality, and medication use (32–35).

② Insomnia Severity Index (ISI): 7-item scale (0–28) (36); scores ≥15 indicate clinically significant insomnia (37); a reduction of ≥8 points indicates meaningful improvement (38).

③ Pittsburgh Sleep Quality Index (PSQI): Global sleep quality (0–21); scores > 5 indicate poor sleep quality (39–42).

(2) *Daytime Functioning Assessment*

① Epworth Sleepiness Scale (ESS): Daytime sleepiness across eight situations (43, 44).

② Fatigue Severity Scale (FSS): 9-item scale (9–63); scores ≥36 indicate clinically significant fatigue (45–47).

③ Generalized Anxiety Disorder 7-item Scale (GAD-7): Anxiety severity (0–21) (48–50).

④ Patient Health Questionnaire-9 (PHQ-9): Depression symptom severity (51, 52).

(3) *Medication Reduction Response Assessment*

① NBZDs Reduction Rate

The NBZDs reduction rate was calculated using the following formula: NBZDs reduction rate = (Pre-treatment weekly NBZDs dose - Post-treatment weekly NBZDs dose) / Pre-treatment weekly NBZDs dose * 100% (Note: Pre-treatment refers to the baseline period, and post-treatment refers to week 6 of the treatment period) (16).

② Benzodiazepine Hypnotics Withdrawal Symptom Scale (BHWSS): 12-item self-report (3-point scale) assessing withdrawal symptoms (53, 54).

③ Medication Craving Visual Analogue Scale (VAS): 0–10 scale (0 = no craving, 10 = extreme craving) (55).

(4) *Objective sleep assessment parameters*

① Polysomnography (PSG): performed as the primary objective sleep assessment, with all participants undergoing one night of 8-h monitoring during screening to exclude other sleep disorders; sleep stages and parameters were manually scored according to the AASM Manual (Version 3.0) (56–58).

② Actigraph (ACT): Recorded for 7 consecutive days at baseline and at the end of intervention; parameters included number of awakenings (NOA), total wake time (TWT), sleep onset latency (SOL), total sleep time (TST), and sleep efficiency (SE) (59–62).

Objective sleep measures (PSG and ACT) were not collected at the 10-week follow-up because the primary focus of this trial was NBZDs discontinuation, not direct sleep improvement. To enhance patient convenience and retention, some participants will be followed up online (video/phone) at week 10, precluding in-person objective measurements. PSG is resource-intensive and burdensome, and actigraphy requires prolonged device wear, both of which could increase dropout rates in this pragmatic feasibility study.

(5) *Assessment of acupuncture treatment expectancy*

The Acupuncture Expectancy Scale (AES) is a four-item instrument rated on a 5-point Likert scale, yielding total scores ranging from 4 to 20 (63). The AES evaluates participants’ pretreatment expectations regarding acupuncture intervention, with higher scores indicating greater expectancy. Scores >12 indicate high expectancy, whereas scores ≤12 indicate low expectancy (64). The AES was administered once prior to treatment initiation.

(6) *Blinding assessment*

Blinding effectiveness will be evaluated by asking participants whether they perceived needle penetration during treatment. Assessments will be conducted at three time points: baseline (after the first treatment session), mid-term (end of week 3), and final (end of week 6, after the last treatment session). Participants will also be asked to guess their group assignment (electroacupuncture, sham, or unsure) and to rate their confidence level. The blinding index will be calculated at each time point. If systematic unblinding is detected, sensitivity analyses will be performed excluding participants who correctly guessed their assignment with high confidence.

### Efficacy evaluation criteria

① Primary Efficacy Outcome Measures

The primary efficacy outcome is the successful discontinuation rate of NBZDs at the end of the 6-week treatment period. Between-group differences in discontinuation rate will be evaluated using regression-based methods appropriate for binary outcomes.

② Secondary Efficacy Outcome Measures

a. Subjective Sleep Efficacy Evaluation

Subjective sleep outcomes, including the Insomnia Severity Index (ISI) and Pittsburgh Sleep Quality Index (PSQI), will be analyzed using longitudinal mixed-effects models incorporating repeated measurements across predefined study time points to estimate treatment effects over time.

b. Objective Sleep Efficacy Assessment

Objective sleep parameters derived from PSG and ACT will be analyzed using longitudinal mixed-effects models to account for within-subject correlation and to evaluate changes across study time points.

c. Daytime Functioning Assessment

Daytime functioning outcomes will be analyzed using longitudinal mixed-effects models to assess treatment effects across time.

d. NBZDs Reduction Response Evaluation

Medication reduction responses, including NBZDs reduction rate, withdrawal symptoms, and craving scores, will be evaluated using regression-based models appropriate to outcome type, with longitudinal modeling applied where repeated measurements are available.

### Safety evaluation

According to the World Health Organization definition, adverse events (AEs) refer to any untoward medical occurrences during interventional treatment, regardless of their causal relationship with the intervention (65). Potential adverse events related to acupuncture included dizziness, nausea, bleeding, subcutaneous hematoma, severe pain, pruritus at needling sites, needle sticking, and needle breakage. Withdrawal-related reactions during NBZDs dose reduction included anxiety, rebound insomnia, headache, palpitations, and excessive sweating. AE severity was graded as mild, moderate, or severe (66); causality was classified as definite, probable, possible, unrelated, or undeterminable. All AEs were documented in the case report form (CRF).

### Sample size

The expected discontinuation efficacy rate for the sham acupuncture group in the sample size calculation was derived from a randomized controlled trial that enrolled 144 long-term benzodiazepine users (21). However, this trial yielded a negative result, which might be attributable to the simultaneous inclusion of both BZDs and NBZDs in the study medication (22). Therefore, only the sham acupuncture group discontinuation rate of 10.83% was adopted from that trial, while the expected successful discontinuation rate for the acupuncture group was based on our pilot study.

A pilot study was conducted at our center prior to the main trial to assess the feasibility of the intervention and to obtain preliminary estimates of effect size. Ten eligible patients with chronic insomnia disorder who had used NBZDs regularly for >6 months received electroacupuncture (three times per week for 6 weeks) combined with the standardized 6-week NBZD tapering protocol (25% dose reduction every week). The successful discontinuation rate was 50.00% (5/10). No serious adverse events were reported. Detailed pilot study results are provided in Supplementary Appendix S2.

Based on expected outcomes and relevant published literature (21), this study hypothesized that after treatment, the discontinuation efficacy rates of NBZDs at 6-week post-treatment for the electroacupuncture group and sham needle and sham electroacupuncture group would be 50.00 and 10.83%, respectively. This study set *α* = 0.05 and 1-*β* = 0.90, employing a two-sided test with equal sample sizes between treatment and control groups. Referencing the sample size estimation formula for comparing efficacy rates between two groups from Sun Zhenqiu’s Medical Statistics, the calculation formula was as follows:


$$n=1641.4/{\left[\frac{\left(u_{\alpha }+u_{\beta }\right)}{\sin^{-1}\sqrt{P_{1}}-\sin^{-1}\sqrt{P_{2}}}\right]}^{2}$$

where $u_{\alpha }$ =1.96 (for α =0.05, two-sided), $u_{\beta }$ =1.282 (for β =0.10), P_1_ = 50.00%, and P_2_ = 10.83%. The calculation yields n = 31.7 ≈ 32 cases. Accounting for dropout rates, the clinically set dropout rate is 20%, resulting in a final sample size of 39 participants per group, totaling 78 participants across both groups.

### Randomization and blinding

#### Randomization

Random allocation was performed using the centralized randomization system of Zhejiang Provincial Hospital of Traditional Chinese Medicine. Eligible participants who met all inclusion criteria were assigned to either the electroacupuncture group or the sham needle plus sham electroacupuncture group in a 1:1 ratio, with 39 participants allocated to each group.

#### Blinding

This study adopts a single-blind design in which participants, outcome assessors, and statistical analysts are blinded to group allocation. Participants are treated in separate rooms to minimize communication. Acupuncturists receive standardized training and follow semi-structured communication procedures to avoid disclosure of group assignment. Outcome assessments are conducted strictly according to predefined case report form procedures. An independent statistician blinded to allocation performs all analyses.

Emergency unblinding will be permitted only when necessary for participant safety. All unblinding procedures require approval from the principal investigator and are fully documented. Participants who undergo emergency unblinding will be excluded from efficacy analyses but retained in safety analyses.

### Data management

Data collection will encompass three aspects: (1) Participants independently completing relevant scale assessments in the acupuncture treatment room. The assessment requires approximately half an hour. During this period, participants may consult dedicated assessment physicians regarding any questions. (2) Participants will complete sleep diaries at home throughout the study period under physician guidance.

This study will train data outcome assessors to enhance participant compliance, collect high-quality complete datasets, and facilitate follow-up completion. Dedicated personnel will perform data entry, receiving specialized training in data input procedures, coding protocols, and secure storage practices. Data analysts in this study will receive training in data evaluation and analysis methodologies. This study strictly safeguards participant privacy through the following primary measures: ① Requiring participants to provide only research-related personal information during the study process; ② Store data on designated computers with restricted access, ensuring the specific folders containing data are password-protected; ③ Only personnel involved in the research can access participants’ personal information, and this information will be destroyed upon completion of the study.

The full analysis set (FAS) will include all randomized participants who receive at least one treatment session and have at least one post-baseline assessment, consistent with the intention-to-treat principle. Missing outcome data will be handled using likelihood-based estimation within mixed-effects models under the missing-at-random assumption. Multiple imputation will be conducted as a sensitivity analysis. The safety set (SS) will include all participants who receive at least one treatment session. The pilot data and the main trial data will be pooled after completion of the main trial to conduct sensitivity analyses assessing the robustness of the conclusions.

### Statistical analysis

All statistical analyses will be conducted using SPSS version 28.0 (IBM Corp., Armonk, NY, United States) according to the intention-to-treat principle. A two-sided *p* < 0.05 will be considered statistically significant.

Continuous variables will be summarized as mean ± standard deviation or median (interquartile range), as appropriate. Categorical variables will be presented as frequencies and percentages. Between-group differences at single time points will be assessed using independent-samples *t*-tests or Mann–Whitney U tests for continuous variables and *χ*^2^ or Fisher’s exact tests for categorical variables.

Longitudinal outcomes will be analyzed using linear mixed-effects models with fixed effects for group, time, and group-by-time interaction, and random intercepts for participants. Missing data will be handled using likelihood-based estimation under the missing-at-random assumption. Multiple imputation will be conducted as a sensitivity analysis.

## Discussion

This randomized, controlled, single-blind trial is designed to evaluate the efficacy and safety of electroacupuncture as an adjunctive intervention to facilitate tapering of NBZDs in patients with CID. The present discussion contextualizes the scientific rationale for the study, outlines the anticipated clinical implications of the findings, and critically appraises key methodological considerations.

### Clinical management of chronic insomnia

Cognitive behavioral therapy for insomnia (CBT-I) is internationally recognized as the first-line treatment for CID. This multimodal intervention encompasses sleep restriction, stimulus control, relaxation training, sleep hygiene education, and cognitive restructuring (67), and has demonstrated efficacy not only in alleviating insomnia symptoms but also in modifying maladaptive sleep-related cognitions and behaviors, thereby promoting durable improvements in sleep regulation (68). However, the implementation of CBT-I in China remains limited due to multiple barriers, including high treatment costs, insufficient availability of trained providers, and the relatively prolonged treatment course (69).

The high prevalence of CID and the widespread reliance on NBZDs, together with the well-documented challenges associated with their long-term use, create a substantial clinical dilemma. Although NBZDs remain a cornerstone of pharmacological management, their dependence potential and withdrawal-related distress constitute major barriers to discontinuation, leaving many patients trapped in a cycle of chronic use. Existing discontinuation strategies—including pharmacological substitution, which carries its own adverse effect profile (12, 13, 15), and educational or behavioral interventions with variable implementation fidelity—have not consistently provided safe and effective solutions (70, 71). This trial addresses this unmet clinical need by evaluating electroacupuncture as a non-pharmacological adjunct to support medication deprescribing. By focusing specifically on NBZDs users, the study targets the most widely prescribed first-line hypnotics, thereby enhancing the direct clinical relevance of its findings.

### The role of acupuncture in tapering NBZDs

The current evidence base regarding acupuncture-assisted medication discontinuation remains limited and heterogeneous. Conflicting findings from prior randomized controlled trials, including the negative results reported by Lao et al. (21) and the positive outcomes described by Zhang et al. (24) underscore the need for methodologically rigorous investigation. The present trial incorporates several design refinements intended to address key limitations of previous studies and provide more definitive evidence.

First, the lack of stratification by benzodiazepine receptor agonist subtype may have confounded prior findings. Although traditional benzodiazepines and NBZDs share overlapping pharmacodynamic properties, they differ in pharmacokinetic profiles and receptor subunit selectivity, factors that may influence withdrawal severity and discontinuation success (72). By restricting enrollment to NBZDs users, the present study enhances population homogeneity, reduces clinical heterogeneity, and increases statistical sensitivity to detect treatment-specific effects.

Second, the challenge of maintaining adequate blinding in acupuncture research is addressed through a rigorous sham-control procedure. The use of validated non-penetrating needles applied at the same acupoints is intended to preserve participant blinding while controlling for non-specific effects associated with needling procedures, including expectancy, attention, and therapeutic context. This design strengthens internal validity and facilitates a more precise evaluation of the specific physiological effects of electroacupuncture.

### Potential mechanisms of acupuncture in tapering NBZDs

The potential therapeutic effects of electroacupuncture in facilitating NBZDs tapering may be considered within converging neurobiological and theoretical frameworks. From a neurobiological perspective, chronic NBZDs exposure induces adaptive changes in the *γ*-aminobutyric acid (GABA)ergic system, including downregulation of GABA_A receptors. Withdrawal disrupts neurochemical equilibrium, producing central nervous system hyperexcitability that manifests as rebound insomnia, anxiety, and other withdrawal symptoms, which in turn increase relapse risk (5, 9). Electroacupuncture has been suggested by some preclinical and clinical studies to potentially modulate central neurotransmitter systems, possibly including promoting GABA synthesis and release, and influencing other systems involved in sleep and arousal, such as the monoaminergic and orexin systems (20, 73). It is hypothesized that by restoring neurochemical homeostasis, electroacupuncture could “bridge” the neuroadaptive gap during NBZDs taper, mitigating withdrawal symptoms and stabilizing sleep architecture, thereby might enabling successful dose reduction.

From the perspective of traditional Chinese medicine (TCM), long-term NBZDs use is conceptualized as disrupting the harmonious flow of Qi and Blood, contributing to Liver Qi stagnation transforming into Fire that disturbs the Heart and destabilizes the Shen (mind-spirit). The resulting internal disequilibrium is considered within TCM theory as a central mechanism underlying persistent insomnia and anxiety during medication withdrawal. The selected partial acupoint prescription—including Hegu (LI4), Taichong (LR3), Neiguan (PC6), Shenmen (HT7), Sanyinjiao (SP6), Baihui (GV20), Sishencong (EX-HN1), Anmian (Extra)—is intended to soothe the Liver, clear pathogenic Fire, nourish the Heart, and calm the Shen. This integrative framework, combining hypothetical neurobiological modulation with TCM-based regulatory principles, provides a potential coherent theoretical rationale for the intervention.

Importantly, the current study was not designed to test any neurobiological or TCM-based mechanisms. All mechanistic interpretations presented above are hypothetical and exploratory in nature, based solely on literature-derived inferences rather than direct experimental evidence from this trial. We did not measure any biomarkers (e.g., GABA, cortisol, or cytokines) nor employ neuroimaging (e.g., fMRI). Future studies incorporating biomarker panels and neuroimaging endpoints are necessary to validate or refute these proposed mechanisms and to identify patient subgroups most likely to benefit from electroacupuncture-assisted NBZDs tapering.

### Potential impact and clinical implications

If electroacupuncture demonstrates efficacy in facilitating NBZDs discontinuation, the findings would have important implications for the management of CID. Electroacupuncture could be incorporated into structured deprescribing programs as a safe, non-pharmacological adjunct, offering a therapeutic option for patients who have failed prior discontinuation attempts, are intolerant to pharmacological alternatives, or prefer non-drug interventions. Successful reduction of NBZDs dependence may also contribute to decreased risks associated with long-term hypnotic use, including falls, cognitive impairment, and healthcare utilization (6, 7).

Conversely, if no significant benefit is observed despite methodological enhancements, the results would provide important negative evidence. Such findings would suggest that previously reported benefits may reflect non-specific effects or that acupuncture alone may be insufficient to overcome neuroadaptive changes associated with NBZDs dependence. This outcome would help refine future research directions and guide the development of alternative or combination-based non-pharmacological strategies.

### Study limitations

Several limitations should be acknowledged. First, the generalizability of our findings may be constrained by the single-center design and the use of a standardized tapering protocol. As an early-stage exploratory study, the single-center design was intentionally adopted to maximize internal validity, minimize the heterogeneity and costs associated with multi-center trials, and enhance reproducibility, thereby laying the groundwork for future multi-center investigations. However, this design inevitably involved a relatively homogeneous and highly adherent patient population, which may introduce selection bias. Additionally, access to specialized acupuncturists and frequent monitoring—resources that are not universally available in routine clinical settings—further limits the applicability of our findings. Furthermore, while the standardized tapering protocol reduced performance bias, improved reproducibility, and provided a clear estimate of treatment efficacy, its rigidity may lead to suboptimal adherence in some patients and fails to account for real-world interpatient variability. Therefore, our results reflect efficacy under optimized conditions; the effectiveness of this intervention in heterogeneous, resource-limited, or less supervised settings may differ. Future pragmatic multi-center trials incorporating flexible, patient-centered tapering strategies are needed to establish broader applicability. Second, the single-blind design ensures participant blinding but does not permit blinding of treating acupuncturists, introducing the possibility of performance bias. This design was selected to preserve treatment fidelity while maintaining methodological rigor. Third, it must be acknowledged that the small sample size of the pilot study may have introduced uncertainty in the estimation of the effect size. Therefore, after completion of the main trial, the pilot data will be pooled with the main trial data to conduct sensitivity analyses assessing the robustness of the conclusions under different effect size assumptions. Furthermore, future multi-center, large-sample confirmatory trials are recommended to validate the findings of the present study. As a final note, although plausible neurobiological mechanisms are proposed, mechanistic pathways are not directly evaluated. Future studies incorporating neuroimaging, electrophysiological measures, or biomarker assessments would help clarify the underlying mechanisms of action.

## Conclusion

In summary, this rigorously designed clinical trial seeks to generate high-quality evidence regarding the role of electroacupuncture as an adjunctive intervention to facilitate discontinuation of NBZDs. By addressing key methodological limitations of previous studies and grounding the intervention in a coherent theoretical framework, the trial is expected to contribute meaningful evidence to the field. The findings will help clarify the clinical value of acupuncture in the management of hypnotic deprescribing and may inform the development of safer, less medication-dependent treatment strategies for patients with chronic insomnia.
