# Supplementary material for: Adjunctive electroacupuncture to facilitate discontinuation of non-benzodiazepine hypnotics in chronic insomnia: a randomized controlled trial protocol
Source: Front Neurol. 2026 May 6;17:1835295. doi: 10.3389/fneur.2026.1835295 (PMC13189547; doi:10.3389/fneur.2026.1835295)
Supplement: Supplementary file 1 [file Supplementary_file_1.docx]

**Table 1s Sociodemographic, hypnotic-use, and baseline clinical characteristics of participants in the pilot study.**

| **Variable** | **All participants n = 10** | **Range** |
| --- | --- | --- |
| Age, y | 41.5 ± 12.2 | 25.0–62.0 |
| Female sex, % | 5 (50.0) | / |
| Han ethnicity, % | 10 (100.0) | / |
| BMI, kg/m² | 23.4 ± 2.9 | 20.3–28.7 |
| **Educational level, %** |  |  |
| Junior middle school or below | 2 (20.0) | / |
| High school/technical secondary school | 3 (30.0) | / |
| College/Bachelor's degree | 4 (40.0) | / |
| Master's degree or above | 1 (10.0) | / |
| **Marital status, %** |  |  |
| Never married | 3 (30.0) | / |
| Married | 5 (50.0) | / |
| Divorced | 1 (10.0) | / |
| Widowed | 1 (10.0) | / |
| **Occupation, %** |  |  |
| Worker | 6 (60.0) | / |
| Housewife | 1 (10.0) | / |
| Student | 1 (10.0) | / |
| Unemployed | 2 (20.0) | / |
| **Coffee drinking habit, %** |  |  |
| None | 6 (60.0) | / |
| <1 time/week | 2 (20.0) | / |
| ≥3 times/week | 2 (20.0) | / |
| **Tea drinking habit, %** |  |  |
| None | 4 (40.0) | / |
| <1 time/week | 5 (50.0) | / |
| ≥3 times/week | 1 (10.0) | / |
| **Hypnotic type, %** |  |  |
| Eszopiclone | 5 (50.0) | / |
| Zolpidem | 5 (50.0) | / |
| **Starting dose, mg/night** |  |  |
| Eszopiclone | 4.2 ± 1.6 | 3.0–6.0 |
| Zolpidem | 8.0 ± 2.7 | 5.0–10.0 |
| **Baseline clinical scales** |  |  |
| ISI | 18.8 ± 2.0 | 15.0–22.0 |
| PSQI | 12.0 ± 2.0 | 9.0–15.0 |
| ESS | 10.1 ± 1.9 | 7.0–13.0 |
| FSS | 4.1 ± 1.1 | 3.0–6.0 |
| GAD-7 | 2.3 ± 1.3 | 0.0–4.0 |
| PHQ-9 | 2.8 ± 0.8 | 2.0–4.0 |
| Craving VAS | 7.9 ± 1.0 | 6.0–9.0 |

Abbreviations: BMI, body mass index; ESS, Epworth Sleepiness Scale; FSS, Fatigue Severity Scale; GAD-7, Generalized Anxiety Disorder-7; ISI, Insomnia Severity Index; PHQ-9, Patient Health Questionnaire-9; PSQI, Pittsburgh Sleep Quality Index; VAS, visual analogue scale. Data are presented as mean ± SD or number (%). BMI was calculated as weight (kg)/height (m)^2.

**Table 2s Medication use and clinical outcomes across study time points in the pilot study.**

|  | **Electroacupuncture pilot cohort (n = 10)** | |
| --- | --- | --- |
| **Outcome / time point** | **Mean (SE) or n (%)** | **Within-group effect size (95% CI)** |
| **Successful discontinuation rate, %** |  |  |
| Week 6 | 5/10 (50.0) | / |
| Follow-up week 1 | 5/10 (50.0) | / |
| Follow-up week 3 | 5/10 (50.0) | / |
| **Eszopiclone dose, mg/night (n = 5)** |  |  |
| Baseline | 4.2 (0.7) |  |
| Week 2 | 3.4 (0.7) | 0.44 (-0.82, 1.69) |
| Week 4 | 1.8 (0.4) | 1.66 (0.18, 3.15) |
| Week 6 | 0.6 (0.4) | 2.46 (0.73, 4.19) |
| Follow-up week 1 | 0.8 (0.5) | 2.20 (0.56, 3.84) |
| Follow-up week 3 | 0.6 (0.4) | 2.46 (0.73, 4.19) |
| **Zolpidem dose, mg/night (n = 5)** |  |  |
| Baseline | 8.0 (1.2) |  |
| Week 2 | 7.0 (0.9) | 0.37 (-0.88, 1.62) |
| Week 4 | 4.6 (0.9) | 1.28 (-0.11, 2.68) |
| Week 6 | 2.1 (1.0) | 2.17 (0.54, 3.80) |
| Follow-up week 1 | 2.5 (1.1) | 1.89 (0.35, 3.44) |
| Follow-up week 3 | 2.5 (1.1) | 1.89 (0.35, 3.44) |
| **Reduction from baseline dose, %** |  |  |
| Week 2 | 15.0 (5.1) | / |
| Week 4 | 47.3 (7.4) | / |
| Week 6 | 79.8 (7.4) | / |
| Follow-up week 1 | 72.5 (11.1) | / |
| Follow-up week 3 | 75.8 (10.3) | / |
| **ISI** |  |  |
| Baseline | 18.8 (0.6) |  |
| Week 2 | 14.9 (0.7) | 1.77 (0.72, 2.82) |
| Week 4 | 11.7 (1.0) | 2.62 (1.39, 3.84) |
| Week 6 | 9.1 (1.2) | 3.13 (1.78, 4.48) |
| Follow-up week 1 | 8.5 (1.3) | 2.97 (1.67, 4.28) |
| Follow-up week 3 | 7.8 (1.1) | 3.66 (2.18, 5.14) |
| **PSQI** |  |  |
| Baseline | 12.0 (0.6) |  |
| Week 6 | 5.2 (0.6) | 3.36 (1.96, 4.77) |
| Follow-up week 3 | 5.5 (0.7) | 2.98 (1.67, 4.29) |
| **ESS** |  |  |
| Baseline | 10.1 (0.6) |  |
| Week 6 | 5.2 (0.6) | 2.41 (1.23, 3.58) |
| Follow-up week 3 | 4.7 (0.7) | 2.53 (1.33, 3.74) |
| **FSS** |  |  |
| Baseline | 4.1 (0.3) |  |
| Week 6 | 3.4 (0.3) | 0.68 (-0.22, 1.59) |
| Follow-up week 3 | 3.2 (0.3) | 0.85 (-0.07, 1.77) |
| **GAD-7** |  |  |
| Baseline | 2.3 (0.4) |  |
| Week 6 | 1.6 (0.4) | 0.52 (-0.38, 1.41) |
| Follow-up week 3 | 1.3 (0.3) | 0.79 (-0.12, 1.71) |
| **PHQ-9** |  |  |
| Baseline | 2.8 (0.2) |  |
| Week 6 | 1.7 (0.4) | 1.01 (0.07, 1.94) |
| Follow-up week 3 | 3.5 (2.2) | -0.14 (-1.01, 0.74) |
| **Craving VAS** |  |  |
| Baseline | 7.9 (0.3) |  |
| Week 2 | 7.9 (0.3) | 0.00 (-0.88, 0.88) |
| Week 4 | 6.0 (0.4) | 1.55 (0.54, 2.56) |
| Week 6 | 2.2 (0.8) | 2.88 (1.60, 4.17) |
| Follow-up week 1 | 2.7 (1.0) | 2.21 (1.08, 3.35) |
| Follow-up week 3 | 2.3 (0.8) | 2.70 (1.46, 3.94) |
| **BHWSS** |  |  |
| Week 2 (n = 5) | 0.4 (0.2) | / |
| Week 4 (n = 7) | 0.9 (0.3) | / |
| Week 6 (n = 7) | 1.3 (0.3) | / |
| Follow-up week 1 (n = 7) | 1.1 (0.3) | / |
| Follow-up week 3 (n = 10) | 0.7 (0.2) | / |

Abbreviations: BHWSS, Benzodiazepine Hypnotics Withdrawal Symptom Scale; ESS, Epworth Sleepiness Scale; FSS, Fatigue Severity Scale; GAD-7, Generalized Anxiety Disorder-7; ISI, Insomnia Severity Index; PHQ-9, Patient Health Questionnaire-9; PSQI, Pittsburgh Sleep Quality Index; VAS, visual analogue scale. Data are presented as mean (SE) or n (%). Within-group effect sizes were calculated versus baseline using Hedges’ g with 95% CIs; positive values indicate improvement relative to baseline. Reduction from baseline dose was calculated as (baseline dose – observed dose)/baseline dose × 100%. BHWSS values were summarized from available assessments only; cells recorded as “/” in the source workbook were treated as not assessed/missing.
