# Supplementary material for: Adjunctive electroacupuncture to facilitate discontinuation of non-benzodiazepine hypnotics in chronic insomnia: a randomized controlled trial protocol
Source: Front Neurol. 2026 May 6;17:1835295. doi: 10.3389/fneur.2026.1835295 (PMC13189547; doi:10.3389/fneur.2026.1835295)
Supplement: Supplementary file 2 [file Supplementary_file_2.docx]

**Methods**

**Outcome Measures**

**Secondary Outcome Measures**

Secondary outcomes will be assessed at baseline and at predefined time points during and after the intervention period, and include the following domains.

(1) Subjective Sleep Scales

① Consensus Sleep Diary (CSD)

The sleep diary is a fundamental tool in insomnia research and is widely regarded as the gold standard for assessing subjective sleep.^[30, 31]^ It primarily captures nocturnal sleep patterns and related daytime conditions. In 2012, the journal Sleep published a seminal article introducing the CSD, establishing it as a standardized prospective self-monitoring instrument for sleep assessment.^[32]^ This clinically practical and effective tool facilitates the evaluation and longitudinal monitoring of treatment effects in insomnia. In the present study, the core version of the CSD was adopted. Recorded variables included total sleep time (TST), sleep onset latency (SOL), wake after sleep onset (WASO), sleep efficiency (SE), subjective sleep quality, and medication use.^[33]^ Subjective sleep quality was rated on a 5-point Likert scale ranging from “very poor” to “very good.” Mean scores for each item were calculated for each participant. Based on the general instructions of the CSD, a sleep diary user manual was developed. Participants received face-to-face training on diary completion to enhance data accuracy and completeness. The CSD was administered at the following time points: pre-treatment, week 2 and week 4 of treatment, week 6 of treatment, week 2 of follow-up, and week 4 of follow-up.

② Insomnia Severity Index (ISI)

The ISI is a 7-item self-report scale designed to assess perceived insomnia severity and its impact on daytime functioning over the preceding two weeks. It is widely used for insomnia screening and for evaluating treatment response.^[34]^ The scale assesses difficulty initiating sleep, difficulty maintaining sleep, early morning awakening, satisfaction with current sleep pattern, interference with daytime functioning, noticeability of sleep problems, and distress caused by sleep difficulties.^[35]^ The total ISI score ranges from 0 to 28, with scores ≥15 indicating clinically significant insomnia. A reduction of ≥8 points is considered indicative of clinically meaningful improvement.^[36]^ The Chinese version of the ISI has demonstrated good reliability and validity for assessing insomnia in Chinese populations.^[37]^ The ISI was administered at pre-treatment, week 2, week 4, and week 6 of treatment, as well as week 2 and week 4 of the follow-up period.

③ Pittsburgh Sleep Quality Index (PSQI)

The PSQI was used as a supplementary instrument to assess nocturnal sleep quality and daytime functioning over the preceding month.^[38]^ The total PSQI score ranges from 0 to 21, with scores >5 indicating clinically significant poor sleep quality.^[39]^ The Chinese version of the PSQI has demonstrated high reliability and validity and is widely used in both clinical practice and research settings.^[40]^ The PSQI was administered at three time points: pre-treatment, week 6 of treatment, and week 4 of the follow-up period.

(2) Daytime Functioning Assessment

① Epworth Sleepiness Scale (ESS)

This instrument evaluates participants’ level of daytime sleepiness. Originally developed by Australian physician Dr. Murray Johns in 1991, the ESS assesses dozing likelihood across eight situational contexts.^[41]^ Higher scores indicate greater propensity for unintended sleep onset. The validated Simplified Chinese version of ESS exhibits excellent psychometric properties including strong validity and reliability.^[42]^ This scale was administered at three time points: before treatment initiation, at week 6 of treatment, and during week 4 of the follow-up period.

② Fatigue Severity Scale (FSS)

The FSS was used to assess the severity of daytime fatigue. The FSS was developed by Krupp et al. in 1989 and consists of 9 items reflecting subjective fatigue over the past week.^[43]^ Total scores range from 9 to 63, with higher scores indicating more severe fatigue. A total score ≥ 36 indicates clinically significant fatigue and suggests impaired daytime functioning.^[44]^ The Chinese version of the FSS has been validated for reliability and validity and is a standardized instrument for fatigue assessment.^[45]^ The FSS was administered at pre-treatment, week 6 of treatment, and week 4 of the follow-up period.

③ Generalized Anxiety Disorder 7-item Scale (GAD-7)

The GAD-7, developed by Spitzer et al., is a brief self-report instrument widely used to screen for generalized anxiety disorder and to quantify anxiety symptom severity over the preceding two weeks.^[46]^ The scale consists of seven items rated on a 4-point Likert scale, yielding total scores ranging from 0 to 21, with higher scores indicating greater anxiety severity.^[47]^ The GAD-7 demonstrates robust psychometric properties and has been extensively validated across clinical and community populations. The Chinese version has shown good reliability and construct validity.^[48]^ In this study, the GAD-7 was administered at pre-treatment, week 6 of treatment, and week 4 of the follow-up period.

④ Patient Health Questionnaire-9 (PHQ-9)

The PHQ-9 is a self-report instrument consisting of nine items based on diagnostic criteria from the Diagnostic and Statistical Manual of Mental Disorders published by the American Psychiatric Association.^[49]^ It is a brief and effective tool for assessing depressive symptom severity and has demonstrated good reliability and validity. The Chinese version of the PHQ-9 has also shown satisfactory psychometric properties.^[50]^ The PHQ-9 was administered at pre-treatment, week 6 of treatment, and week 4 of the follow-up period.

(3) Medication Reduction Response Assessment

① NBZDs Reduction Rate

The NBZDs reduction rate was calculated using the following formula: NBZDs reduction rate = (Pre-treatment weekly NBZDs dose - Post-treatment weekly NBZDs dose) / Pre-treatment weekly NBZDs dose * 100%. (Note: Pre-treatment refers to the baseline period, and post-treatment refers to week 6 of the treatment period)^[16]^）. Participants completed daily medication diary cards documenting medication status, including date, medication name, dosage, timing of administration, and adverse reactions. Prescription records were returned to physicians during the first weekly visit and independently verified by observers every three weeks. Assessments were conducted at pre-treatment, week 2, week 4, and week 6 of treatment, and week 4 of the follow-up period.

② Benzodiazepine Hypnotics Withdrawal Symptom Scale (BHWSS)

The Benzodiazepine Hypnotics Withdrawal Symptom Scale (BHWSS) is a self-report questionnaire developed by Kazuo Mishima et al. in 2021 to assess key symptoms experienced during benzodiazepine hypnotic dose reduction.^[51]^ The scale consists of 12 items across five dimensions: perceptual and sensory disturbances, somatic symptoms, depressed mood, involuntary movement control problems (including seizures), and memory impairment. Each item is rated on a 3-point scale. The questionnaire requires less than 10 minutes to complete and is suitable for repeated administration during medication tapering. Validation studies have demonstrated adequate reliability and validity for assessing withdrawal symptom severity following benzodiazepine receptor agonist discontinuation.^[52]^ The BHWSS was administered at pre-treatment, week 2, week 4, and week 6 of treatment, and week 2 and week 4 of the follow-up period.

③ Medication Craving Visual Analogue Scale (VAS)

Medication craving was assessed using a VAS. This unidimensional single-item scale consists of a straight line with two endpoints, where 0 indicates no craving for NBZDs and 10 indicates extreme craving.^[53]^ Participants marked the point on the line that best represented their current craving intensity. Higher scores reflect greater psychological craving for NBZDs. The VAS was administered at pre-treatment, week 2, week 4, and week 6 of treatment, and week 2 and week 4 of the follow-up period.

(4) Objective sleep assessment parameters

① Polysomnography (PSG)

PSG served as the primary objective sleep assessment method in this study. Previous research has shown that NBZDs alter sleep architecture by increasing N2 sleep duration while reducing N3 and N4 sleep, and that long-term use is associated with reduced rapid eye movement (REM) sleep, which may be reversible following discontinuation.^[54]^ All participants underwent PSG during the screening period to exclude other sleep disorders. The PSG system recorded multiple physiological parameters, including electroencephalography (EEG), submental electromyography (EMG), bilateral electrooculography (EOG), oronasal airflow, and bilateral anterior tibialis EMG.^[55]^ Each participant completed one night of PSG monitoring during the screening phase, starting at their habitual bedtime and lasting for 8.0 hours. Sleep EEG data were recorded in 30-second epochs, and all sleep stages and parameters were manually scored in accordance with the AASM Manual for the Scoring of Sleep and Associated Events (Version 3.0).^[56]^ Objective PSG parameters included total sleep time (TST), REM sleep latency (REM-sl), sleep onset latency (SOL), wake after sleep onset (WASO), sleep efficiency (SE), arousal index (ArI), frequency of awakenings (FW), and the percentage of total sleep time spent in each sleep stage.

② Actigraph (ACT)

Actigraphy (ACT) was used as the core objective tool for sleep assessment. This device indirectly determines sleep–wake states by monitoring body movement and is commonly used in clinical practice for objective sleep evaluation. In this study, the MotionWatch 8 actigraph was used. The device has an acceleration sensitivity of < 0.01 g and uses a 5-second epoch, allowing accurate capture of limb movement signals and conversion into sleep-related parameters.^[57, 58]^ The accompanying software was used for offline analysis of sleep and activity data, generating key sleep–wake and rest–activity parameters, including number of awakenings (NOA), total wake time (TWT), sleep onset latency (SOL), total sleep time (TST), and sleep efficiency (SE). The validity of this device has been well established in insomnia populations, with studies demonstrating good agreement with polysomnography (PSG) for sleep–wake assessment (correlation coefficients Rs = 0.52–0.71).^[59]^ As an outcome measure for insomnia treatment efficacy, actigraphy shows good sensitivity, is easy to operate, and is well tolerated by participants, making it suitable for long-term home-based monitoring. Measurements were conducted at baseline and at the end of the intervention, with continuous monitoring for 1 week at each time point. Participants were instructed to maintain their usual daily routines during monitoring and to complete sleep diaries concurrently, ensuring complementary validation between objective and subjective data.^[60]^.

(5) Assessment of Acupuncture Treatment Expectancy

The Acupuncture Expectancy Scale (AES) is a four-item instrument rated on a 5-point Likert scale, yielding total scores ranging from 4 to 20.^[61]^ The AES evaluates participants’ pretreatment expectations regarding acupuncture intervention, with higher scores indicating greater expectancy. Scores >12 indicate high expectancy, whereas scores ≤12 indicate low expectancy.^[62]^ The AES was administered once prior to treatment initiation.

(6) Blinding Assessment:

Blinding effectiveness was evaluated by asking participants whether they perceived needle penetration during treatment. Assessments were conducted at both the initial and final treatment sessions.

**Efficacy Evaluation Criteria**

① Primary Efficacy Outcome Measures

The primary efficacy outcome is the successful discontinuation rate of NBZDs at the end of the 6-week treatment period. Successful discontinuation is defined as complete cessation of NBZDs without relapse to regular use during the final week of treatment, as confirmed by medication records and participant self-reports. Between-group differences in discontinuation rate will be evaluated using regression-based methods appropriate for binary outcomes.

②Secondary Efficacy Outcome Measures

a. Subjective Sleep Efficacy Evaluation

Subjective sleep outcomes, including the Insomnia Severity Index (ISI) and Pittsburgh Sleep Quality Index (PSQI), will be analyzed using longitudinal mixed-effects models incorporating repeated measurements across predefined study time points to estimate treatment effects over time.

b. Objective Sleep Efficacy Assessment

Objective sleep parameters derived from PSG and ACT will be analyzed using longitudinal mixed-effects models to account for within-subject correlation and to evaluate changes across study time points.

c. Daytime Functioning Assessment

Daytime functioning outcomes will be analyzed using longitudinal mixed-effects models to assess treatment effects across time.

d. NBZDs Reduction Response Evaluation

Medication reduction responses, including NBZDs reduction rate, withdrawal symptoms, and craving scores, will be evaluated using regression-based models appropriate to outcome type, with longitudinal modeling applied where repeated measurements are available.

**Safety Evaluation**

According to the World Health Organization definition, adverse events (AEs) refer to any untoward medical occurrences during interventional treatment, regardless of their causal relationship with the intervention.^[63]^ Potential adverse events related to acupuncture included dizziness, nausea, bleeding, subcutaneous hematoma, severe pain, pruritus at needling sites, needle sticking, and needle breakage. Withdrawal-related reactions during NBZDs dose reduction included anxiety, rebound insomnia, headache, palpitations, and excessive sweating.

Adverse event severity was classified using a three-level grading system: mild (well tolerated, requiring no treatment and not affecting normal functioning), moderate (causing some discomfort or requiring intervention with partial impact on normal functioning), and severe (requiring systemic treatment or significantly impairing normal functioning).^[64]^ The relationship between adverse events and the intervention was categorized as definitely related, probably related, possibly related, unrelated, or undeterminable. All adverse events were recorded in the case report form (CRF).

**Reference**

[30] Harris AL, Carmona NE, Moss TG, et al. Testing the contiguity of the sleep and fatigue relationship: a daily diary study[J]. Sleep, 2021, 44(5).

[31] Looman MI, Blanken TF, Schoenmakers TM, et al. Telephone-Guided Sleep Restriction for Insomnia: A Randomized Sleep Diary-Controlled Trial[J]. Psychother Psychosom, 2025, 94(3):147-161.

[32] Carney CE, Buysse DJ, Ancoli-Israel S, et al. The consensus sleep diary: standardizing prospective sleep self-monitoring[J]. Sleep, 2012, 35(2):287-302.

[33] Staiano W, Callahan C, Davis M, et al. Assessment of an App-Based Sleep Program to Improve Sleep Outcomes in a Clinical Insomnia Population: Randomized Controlled Trial[J]. JMIR Mhealth Uhealth, 2025, 13:e68665.

[34] Marway OS, Lau PH, Carmona NE, et al. What are patients completing Cognitive Behavioral Insomnia Therapy telling us with their post-treatment Insomnia Severity Index scores?[J]. Sleep Med, 2023, 103:187-194.

[35] Manzar MD, Jahrami HA, Bahammam AS. Structural validity of the Insomnia Severity Index: A systematic review and meta-analysis[J]. Sleep Med Rev, 2021, 60:101531.

[36] Zhou ES, Ritterband LM, Bethea TN, et al. Effect of Culturally Tailored, Internet-Delivered Cognitive Behavioral Therapy for Insomnia in Black Women: A Randomized Clinical Trial[J]. JAMA Psychiatry, 2022, 79(6):538-549.

[37] Yu DS. Insomnia Severity Index: psychometric properties with Chinese community-dwelling older people[J]. J Adv Nurs, 2010, 66(10):2350-2359.

[38] Olagunju AT, Bioku AA, Olagunju TO, et al. Psychological distress and sleep problems in healthcare workers in a developing context during COVID-19 pandemic: Implications for workplace wellbeing[J]. Prog Neuropsychopharmacol Biol Psychiatry, 2021, 110:110292.

[39] Backhaus J, Junghanns K, Broocks A, et al. Test-retest reliability and validity of the Pittsburgh Sleep Quality Index in primary insomnia[J]. J Psychosom Res, 2002, 53(3):737-740.

[40] Guo S, Sun W, Liu C, et al. Structural Validity of the Pittsburgh Sleep Quality Index in Chinese Undergraduate Students[J]. Front Psychol, 2016, 7:1126.

[41] Kendzerska TB, Smith PM, Brignardello-Petersen R, et al. Evaluation of the measurement properties of the Epworth sleepiness scale: a systematic review[J]. Sleep Med Rev, 2014, 18(4):321-331.

[42] Zhang JN, Peng B, Zhao TT, et al. Modification of the Epworth Sleepiness Scale in Central China[J]. Qual Life Res, 2011, 20(10):1721-1726.

[43] Krupp LB, LaRocca NG, Muir-Nash J, et al. The fatigue severity scale. Application to patients with multiple sclerosis and systemic lupus erythematosus[J]. Arch Neurol, 1989, 46(10):1121-1123.

[44] Tang WK, Chen YK, Liang HJ, et al. Subcortical white matter infarcts predict 1-year outcome of fatigue in stroke[J]. BMC Neurol, 2014, 14:234.

[45] Wang MY, Liu IC, Chiu CH, et al. Cultural adaptation and validation of the Chinese version of the Fatigue Severity Scale in patients with major depressive disorder and nondepressive people[J]. Qual Life Res, 2016, 25(1):89-99.

[46] Spitzer RL, Kroenke K, Williams JB, et al. A brief measure for assessing generalized anxiety disorder: the GAD-7[J]. Arch Intern Med, 2006, 166(10):1092-1097.

[47] Çınar Tanrıverdi E, Yılmaz S, Çayır Y. Evaluation the validity and reliability of the perceived medical school stress scale in Turkish medical students[J]. PLoS One, 2023, 18(8):e0288769.

[48] Shih YC, Chou CC, Lu YJ, et al. Reliability and validity of the traditional Chinese version of the GAD-7 in Taiwanese patients with epilepsy[J]. J Formos Med Assoc, 2022, 121(11):2324-2330.

[49] Huang FY, Chung H, Kroenke K, et al. Using the Patient Health Questionnaire-9 to measure depression among racially and ethnically diverse primary care patients[J]. J Gen Intern Med, 2006, 21(6):547-552.

[50] Cheng J, Ren Z, Rehman S, et al. Comparative assessment of Patient Health Questionnaire-9 and Edinburgh Postnatal Depression Scale for screening antenatal depression among Chinese pregnant women[J]. J Affect Disord, 2025, 376:104-112.

[51] Narisawa H, Inoue Y, Kobayashi M, et al. Development and validation of the Benzodiazepine Hypnotics Withdrawal Symptom Scale (BHWSS) based on item response theory[J]. Psychiatry Res, 2021, 300:113900.

[52] Inoue Y, Takaesu Y, Koebis M. Prevalence of and factors associated with acute withdrawal symptoms after 24 weeks of eszopiclone treatment in patients with chronic insomnia: a prospective, interventional study[J]. BMC Psychiatry, 2021, 21(1):193.

[53] Mehta DD, Sloan ME, Sas A, et al. Craving Induction and Treatment Response in Transcranial Magnetic Stimulation for Tobacco Use Disorder[J]. Biol Psychiatry Cogn Neurosci Neuroimaging, 2026, 11(2):250-256.

[54] Endo H, Shigetsura Y, Tsurumi K, et al. Effects of Dose Reduction or Discontinuation of Benzodiazepine Hypnotics on Sleep and Anxiety in Patients With Insomnia After Long-Term Use[J]. Cureus, 2025, 17(1):e77936.

[55] Wang S, Wu M, Wu S, et al. A polysomnographic study of slow-wave sleep loss in elderly patients with epilepsy[J]. Heliyon, 2024, 10(4):e25904.

[56] Levendowski DJ, Ferini-Strambi L, Gamaldo C, et al. The Accuracy, Night-to-Night Variability, and Stability of Frontopolar Sleep Electroencephalography Biomarkers[J]. J Clin Sleep Med, 2017, 13(6):791-803.

[57] Ran C, Jennysdotter Olofsgård F, Steinberg A, et al. Patients with cluster headache show signs of insomnia and sleep related stress: results from an actigraphy and self-assessed sleep study[J]. J Headache Pain, 2023, 24(1):114.

[58] Pieters LE, Deenik J, Hoogendoorn AW, et al. Sleep and physical activity patterns in relation to daily-life symptoms in psychosis: An actigraphy and experience sampling study[J]. Psychiatry Res, 2025, 344:116320.

[59] Sadeh A. The role and validity of actigraphy in sleep medicine: an update[J]. Sleep Med Rev, 2011, 15(4):259-267.

[60] Landry GJ, Falck RS, Beets MW, et al. Measuring physical activity in older adults: calibrating cut-points for the MotionWatch 8(©)[J]. Front Aging Neurosci, 2015, 7:165.

[61] Gong Y, Li J, Ma HL, et al. High acupuncture expectancy is associated with shorter time to ovulation and higher chances of ovulation in infertile patients with PCOS receiving acupuncture: a secondary analysis of a randomized controlled trial[J]. Acupunct Med, 2025, 43(3):137-143.

[62] Li X, Baser RE, Bryl K, et al. How does pretreatment expectancy influence pain outcomes with electroacupuncture and battlefield acupuncture in cancer survivors?: Pretreatment expectancy and pain reduction by acupuncture[J]. Integr Med Res, 2024, 13(2):101040.

[63] Huang CC, Kotha P, Tu CH, et al. Acupuncture: A Review of the Safety and Adverse Events and the Strategy of Potential Risk Prevention[J]. Am J Chin Med, 2024, 52(6):1555-1587.

[64] Bansal B, Chaturvedi K. Advancing Safety in Acupuncture: Strategies, Best Practices, and Training for a Risk-Free Practice[J]. Med Acupunct, 2025, 37(4):327-338.
